# Association of a Novel Echocardiographic Artificial Intelligence Model With Invasive Hemodynamics to Automate Detection of Heart Failure With Preserved Ejection Fraction

**DOI:** 10.1016/j.shj.2026.101120

**Published:** 2026-09-03

**Authors:** Nadira Hamid, Ashley Akerman, Marat Fudim, Michal Laufer-Perl, Inés Toranzo Nieto, Fawaz Alenezi, Ross Upton, Tamaz Shaburishvili

**Affiliations:** a Division of Cardiology, Allina Health Minneapolis Heart Institute, Minneapolis, Minnesota, USA; b Ultromics Ltd., Oxford, UK; c Division of Cardiology, Department of Medicine, Duke University, Durham, North Carolina, USA; d Tel Aviv Sourasky Medical Center, Affiliated to the School of Medicine, Tel Aviv University, Tel Aviv, Israel; e Instituto de Investigación Biomédica de Salamanca (IBSAL), Salamanca, Spain; f Division of Cardiology, Department of Medicine, Tbilisi Heart and Vascular Clinic, Tbilisi, Georgia

**Keywords:** Artificial intelligence, Heart failure, Interatrial shunt, Preserved ejection fraction

Heart failure (HF) affects up to 1 in 4 adults in their lifetime; half of these patients are classified as HF with preserved ejection fraction (HFpEF).^1^ HFpEF is a highly heterogenous syndrome, making diagnosis complex without invasive hemodynamic assessment.^2^ Artificial intelligence (AI) applied to transthoracic echocardiograms (TTE) has demonstrated excellent discrimination of HFpEF, more often than clinical scores.^3^ Although initial validation of such models have shown promise, their correlation with gold standard invasive hemodynamics has yet to be evaluated.^4^ This pilot study examined the association between AI-based echocardiographic HFpEF models and invasive exercise hemodynamics before and after interatrial shunting, lending insight into the utility of AI-based echo models for improved diagnostic accuracy, HFpEF phenotyping, and trial screening.

Data were pooled from open-label, nonrandomized studies on the safety and feasibility of the Alleviant no-implant interatrial shunt system (Alleviant Medical, Austin, Texas) (ClinicalTrials.gov NCT: 04583527, 04838353, and 05501652). A detailed description of these studies has been published previously.^5^ Briefly, the studies recruited patients with New York Heart Association (NYHA) functional class II through ambulatory IV, left ventricular ejection fraction (LVEF) ≥ 40%, and elevated pulmonary capillary wedge pressure (PCWP) during supine exercise ergometry (≥ 25 mmHg). If hemodynamic criteria were met, subjects underwent the interatrial shunt procedure, whereby a 7-mm atrial septectomy was created in the fossa ovalis using radiofrequency (RF) technology (Alleviant Medical). Follow-up exercise right heart catheterization (RHC) was performed at 1 month after shunt creation with AI-based assessment of TTE at both time points using a previously published model (EchoGo Heart Failure v2.0, Ultromics Ltd, Oxford, UK).^3^

Categorical classification based on probabilistic outputs (positive, negative, or uncertain) and continuous prediction probabilities from the AI HFpEF model were compared with hemodynamic, echocardiographic, patient qualitative, and clinical functional parameters. Model diagnostic performance is summarized as accuracy in HFpEF patients before and after shunt creation. Classification performance assessments do not account for intermediate/uncertain outputs. Pearson correlation coefficient (R) expressed the association between continuous model outputs, hemodynamics, and patient functional status. Continuous variables are summarized using mean ± SD. All analyses were performed using R version 4.2.3 (R Foundation, Vienna, Austria) using a 2-tailed *p*-value of <0.05 for significance. Bootstrap derived 95% CIs are provided where appropriate. As an exploratory pilot analysis, no formal sample-size or power calculation was performed, and the correlation analyses are hypothesis-generating and were not corrected for multiple comparisons. Baseline and 1-month observations were obtained from the same 29 patients and were analyzed as paired, within-patient correlations rather than as independent samples.

Of the 33 available patients, 4 were not analyzed by the AI model given the absence of apical 4 chamber images (n = 1), missing ultrasound region of interest (n = 2), and missing follow-up data (n = 1). Characteristics of the remaining 29 patients were similar to previous reports.^5^ The AI HFpEF model classification for positive, negative, and uncertain changed marginally at baseline compared with 1-month (19, 3, 7 vs. 19, 7, 3, respectively), which was also observed for the H2FPEF score (7, 1, 21 vs. 7, 0, 22, respectively). Before shunt creation, AI accuracy was 86% (95% CI: 68, 100). In 20 patients who had “manifest” HFpEF (rest PCWP ≥ 15 mmHg), accuracy was 82% (95% CI: 58, 100). In contrast, the AI HFpEF model was 100% accurate in 9 patients with “occult” HFpEF (rest PCWP < 15 mmHg, exercise PCWP ≥ 25 mmHg). One month following shunt creation, PCWP decreased by −2.4 mmHg at rest (*p* = 0.065, n = 29), and −7.4 mmHg during exercise (*p* < 0.001, n = 29), and was associated with a modest decrease in AI prediction probability (74% ± 26% to 67% ± 32%; *p* = 0.256).

Correlation statistics are presented in Table 1. Overall, there were moderate to strong associations with echocardiographic parameters consistent with the diagnosis of HFpEF, moderate to strong negative associations with functional parameters, but only weak to moderate associations with invasive hemodynamic and laboratory measures, including PCWP, pulmonary vascular resistance (PVR), and N-terminal pro–B-type natriuretic peptide with CIs that crossed zero (Table 1).

**Table 1:** Correlation statistics.

Parameters	All HFpEF (n = 29)	Manifest HFpEF (n = 20)	Occult HFpEF (n = 9)
PCWP rest			
Baseline	0.144 (−0.235, 0.485)	0.178 (−0.287, 0.575)	0.153 (−0.569, 0.742)
1 mo	0.223 (−0.157, 0.545)	0.246 (−0.221, 0.621)	0.004 (−0.662, 0.667)
PCWP exercise			
Baseline	0.163 (−0.217, 0.499)	0.188 (−0.277, 0.582)	0.081 (−0.616, 0.707)
1 mo	0.213 (−0.166, 0.538)	0.206 (−0.260, 0.594)	−0.028 (−0.679, 0.648)
PVR rest			
Baseline	−0.264 (−0.575, 0.144)	−0.305 (−0.658, 0.159)	−0.111 (−0.722, 0.597)
1 mo	−0.054 (−0.413, 0.319)	0.008 (−0.436, 0.449)	−0.183 (−0.755, 0.548)
PVR exercise			
Baseline	0.180 (−0.251, 0.551)	0.322 (−0.187, 0.695)	−0.082 (−0.838, 0.782)
1 mo	−0.184 (−0.554, 0.247)	−0.348 (−0.710, 0.159)	−0.142 (−0.855, 0.757)
Mean E/e’			
Baseline	0.309 (−0.065, 0.607)	0.207 (−0.259, 0.595)	0.734 (0.137, 0.940)
1 mo	0.556 (0.239, 0.767)	0.594 (0.205, 0.821)	0.648 (−0.029, 0.917)
LA volume index			
Baseline	0.582 (0.274, 0.782)	0.683 (0.345, 0.864)	0.269 (−0.481, 0.792)
1 mo	0.243 (−0.136, 0.560)	0.223 (−0.244, 0.606)	0.253 (−0.494, 0.785)
Global longitudinal strain			
Baseline	0.094 (−0.297, 0.457)	0.081 (−0.401, 0.528)	0.121 (−0.590, 0.727)
1 mo	0.665 (0.358, 0.843)	0.644 (0.237, 0.859)	0.745 (−0.019, 0.960)
LA reservoir strain			
Baseline	0.445 (0.094, 0.698)	0.476 (0.042, 0.759)	0.441 (−0.315, 0.855)
1 mo	0.462 (0.107, 0.712)	0.322 (−0.141, 0.669)	0.635 (−0.127, 0.925)
Six-minute walk test			
Baseline	−0.432 (−0.689, −0.078)	−0.388 (−0.709, 0.066)	−0.600 (−0.904, 0.107)
1 mo	−0.404 (−0.671, −0.043)	−0.677 (−0.862, −0.335)	−0.168 (−0.558, 0.749)
KCCQ			
Baseline	−0.444 (−0.697, −0.093)	−0.378 (−0.703, 0.077)	−0.712 (−0.934, −0.092)
1 mo	−0.053 (−0.412, 0.319)	−0.111 (−0.528, 0.348)	−0.128 (−0.730, 0.586)
NT-proBNP			
Baseline	0.167 (−0.213, 0.503)	0.103 (−0.356, 0.522)	0.636 (−0.049, 0.914)
1 mo	0.204 (−0.175, 0.531)	0.191 (−0.275, 0.584)	0.180 (−0.550, 0.754)

Notes: Pearson coefficients are presented for AI HFpEF model continuous output and clinical parameter associations. Statistics are presented for All HFpEF, Manifest HFpEF, and Occult HFpEF. Model predictions relate to prediction class probability, irrespective of associated uncertainty metrics.

Abbreviations: E/e′, ratio of early mitral inflow velocity (E) to early diastolic mitral annular velocity (e′); HFpEF, heart failure with preserved ejection fraction; KCCQ, Kansas City Cardiomyopathy Questionnaire; LA, left atrial; NT-proBNP, N-terminal pro–B-type natriuretic peptide; PCWP, pulmonary capillary wedge pressure; PVR, pulmonary vascular resistance.

The current pilot study is the first to evaluate a novel echocardiography-based AI model in hemodynamically diagnosed HFpEF patients, and to consider its role in early detection, screening, and identifying patients for optimal therapy. The AI HFpEF model demonstrated high categorical accuracy relative to invasive exercise hemodynamics and, importantly, accurately identified patients who would otherwise require provocation to elicit an increase in filling pressure. Although not specifically developed for this purpose, results indicate that the model is sensitive to invasive and noninvasive estimates and parameters associated with left ventricular and left atrial filling mechanics at rest and during exercise.

The patient cohort represents a unique opportunity to examine how diagnostic models change when an intervention alters the underlying pathophysiology. Interatrial shunt creation improved hemodynamics and functional parameters, but concurrent changes in the average AI model continuous output were not overtly apparent. This might reflect a closer association between the AI model output and noninvasive measurements as expected given the training of the model. More importantly, the minimal change in AI output despite a significant fall in exercise PCWP suggests that the model may principally reflect the chronic structural and functional HFpEF phenotype, including atrial remodeling, myocardial mechanics, and diastolic function, rather than the acute loading state. Because the model is echocardiography derived, its stronger correlations with echocardiographic parameters than with invasive hemodynamics are expected and partly reflect shared measurement. The nonsignificant correlations with PCWP, PVR, and N-terminal pro–B-type natriuretic peptide indicate that the continuous output does not track invasive filling pressures on a per-patient basis. Larger studies which include HFpEF negative controls may help to improve and validate the utility of an AI HFpEF model as a diagnostic aid. The present analysis is limited by a small pilot sample size (n = 29), without a formal power calculation, multiple unadjusted correlations, paired within-patient measurements, a single 1-month follow-up, and the absence of negative controls; the findings should therefore be considered hypothesis generating.

In conclusion, these pilot findings demonstrate that the AI model was accurate in detecting HFpEF, even in patients requiring provocation to elicit increases in filling pressure. Future research will extend these hypothesis-generating findings and examine whether such a model could aid in the diagnosis and screening of HFpEF patients for device therapies.

## Ethics Statement

This analysis pooled data from 3 prospective studies (NCT04583527, NCT04838353, and NCT05501652) that were conducted in accordance with the Declaration of Helsinki and approved by the appropriate institutional review boards or ethics committees. Written informed consent was obtained from all participants.

## Funding

The authors have no funding to report.

## Disclosure Statement

Nadira Hamid reports consulting fees from Abbott Structural, 4C Medical, Philips Healthcare, GE, Siemens, Edwards Lifesciences, Vdyne and Laza. Marat Fudim is a consultant to Alleviant. Ashley Akerman and Ross Upton are employees of Ultromics Ltd.

The other authors had no conflicts to declare.
